# Developmental programming of tissue-resident macrophages

**DOI:** 10.3389/fimmu.2024.1475369

**Published:** 2024-11-07

**Authors:** Maria Francesca Viola, Eliana Franco Taveras, Elvira Mass

**Affiliations:** Developmental Biology of the Immune System, Life and Medical Sciences (LIMES) Institute, University of Bonn, Bonn, Germany

**Keywords:** macrophage, niche, developmental programming, maternal immune activation (MIA), hematopoiesis

## Abstract

Macrophages are integral components of the innate immune system that colonize organs early in development and persist into adulthood through self-renewal. Their fate, whether they are replaced by monocytes or retain their embryonic origin, depends on tissue type and integrity. Macrophages are influenced by their environment, a phenomenon referred to as developmental programming. This influence extends beyond the local tissue microenvironment and includes soluble factors that can reach the macrophage niche. These factors include metabolites, antibodies, growth factors, and cytokines, which may originate from maternal diet, lifestyle, infections, or other developmental triggers and perturbations. These influences can alter macrophage transcriptional, epigenetic, and metabolic profiles, affecting cell-cell communication and tissue integrity. In addition to their crucial role in tissue immunity, macrophages play vital roles in tissue development and homeostasis. Consequently, developmental programming of these long-lived cells can modulate tissue physiology and pathology throughout life. In this review, we discuss the ontogeny of macrophages, the necessity of developmental programming by the niche for macrophage identity and function, and how developmental perturbations can affect the programming of macrophages and their subtissular niches, thereby influencing disease onset and progression in adulthood. Understanding these effects can inform targeted interventions or preventive strategies against diseases. Finally, understanding the consequences of developmental programming will shed light on how maternal health and disease may impact the well-being of future generations.

## 1 Introduction

**Figure 1:**
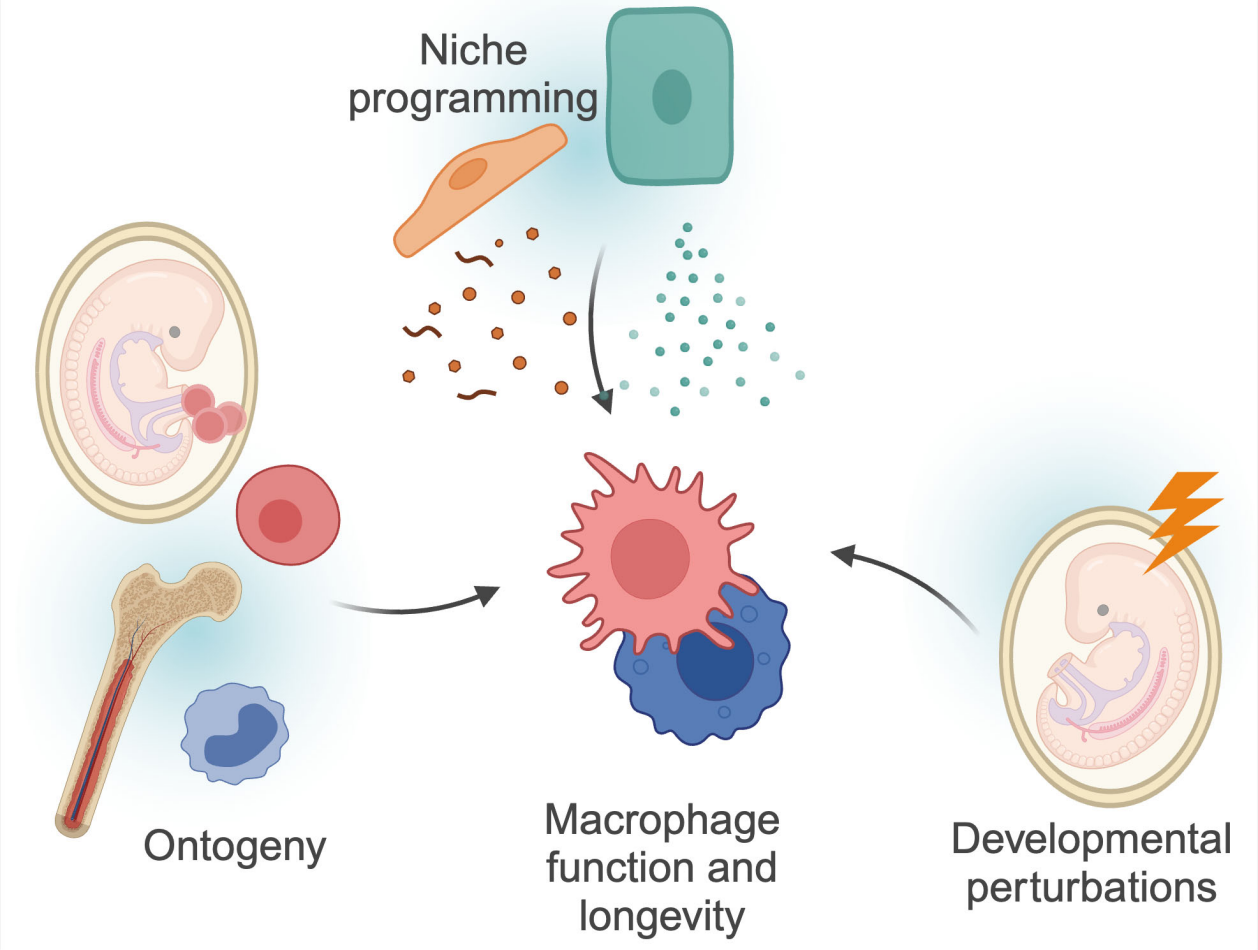
Factors determining macrophage functions. Macrophage function and longevity is a product of numerous, factors. Macrophages can derive from progenitors seeded within the embryo, or from differentiation of circulating monocytes. These cells are then further instructed by local cues and instructions from surrounding cells, the so-called macrophage ‘niche’. Finally, as discussed in more detail in this review, macrophages can be programmed by triggers during development, and retain this information throughout their lifespan. These factors are interconnected; for example, niche perturbations can impact monocyte recruitment, and developmental programming may also impact niche programming itself. Created using BioRender.

Tissue-resident macrophages are integral components of the innate immune system, ubiquitously present across all organs. Initially described by Nobel laureate Ilya Metchnikov as professional phagocytes, macrophages play a crucial role in the clearance of invading pathogens, apoptotic corpses, immune complexes and other cellular debris, thereby maintaining tissue homeostasis. Over the years, our understanding of macrophages has evolved beyond their role as mere ‘garbage cleaners’ to recognizing their vital, tissue-specific functions (1). These specialized roles are directed by signals from the surrounding cells within the tissue, collectively known as the ‘macrophage niche,’ which provides cues that tailor macrophage activity to meet local requirements. Additionally, macrophage function is likely influenced by their ontogeny and the factors and stimuli encountered during early life (**Figure 1**). Here, we review the current knowledge on the developmental programming of tissue-resident macrophages, and how this imprinting may affect tissue development and homeostasis.

## 2 Macrophage ontogeny and longevity

**Figure 2:**
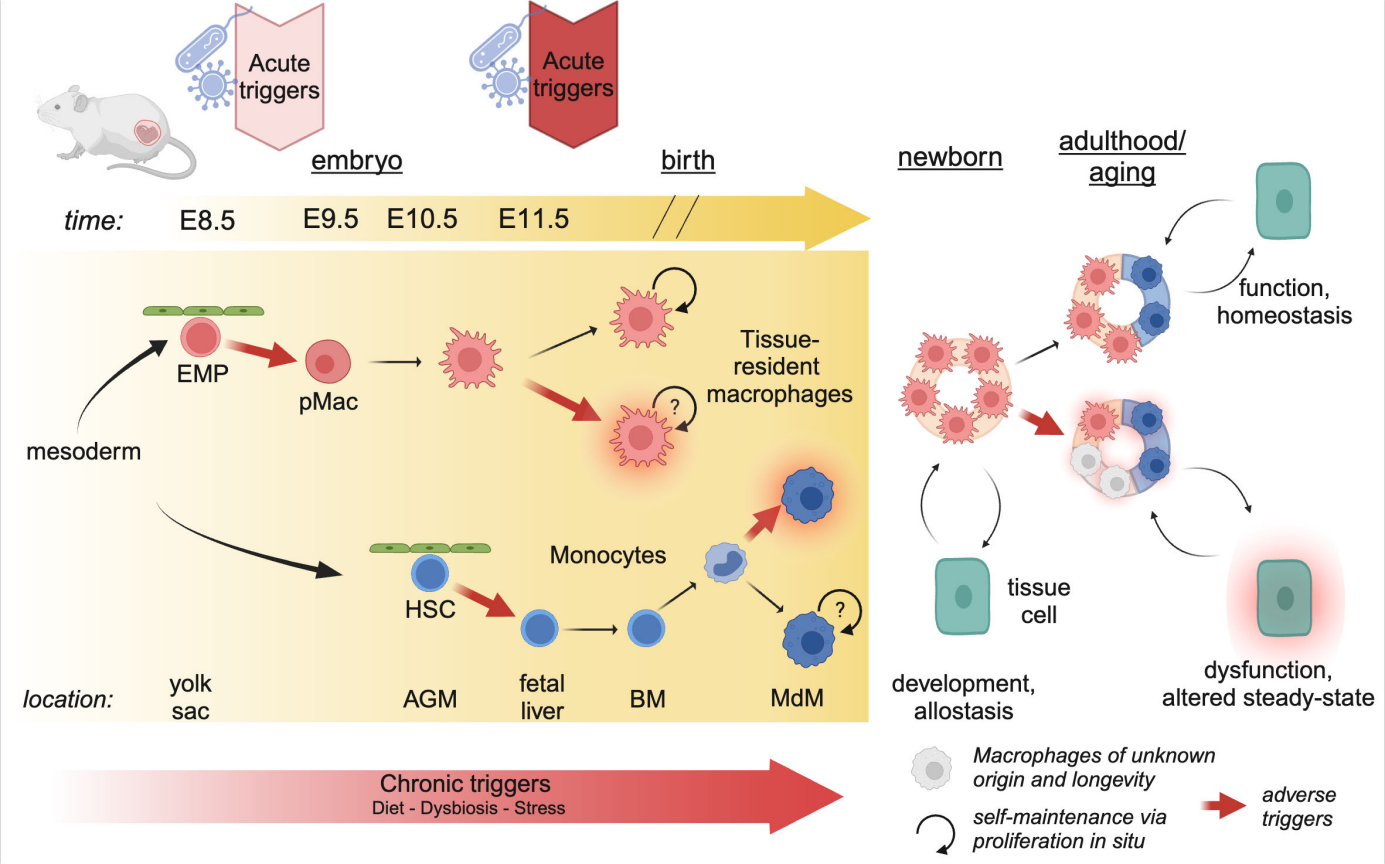
Developmental perturbations during hematopoiesis. The progenitors of tissue-resident macrophages develop in a series of waves during embryogenesis. As early as E8.5, erythro-myeloid progenitors (EMP) in the yolk sac give rise to pre-Macrophages (pMacs), which colonize the developing tissues and give rise to long-lived, proliferating tissue-resident macrophages. At E10.5, hematopoietic stem cells (HSCs) leave the aorta-gonado-mesonephros (AGM) region and migrate to the fetal liver. Here they expand, before migrating to the bone-marrow (BM) just before birth. In postnatal mice, bone-marrow derived monocytes can enter the tissue and differentiate into macrophages (MdM), possibly with different life cycles. The staggered timing of these developmental waves underlies the differential impact triggers can have. Acute triggers, depending on their timing, may program EMP/pMacs and/or HSCs, while chronic triggers may affect all lineages giving rise to macrophages. Created using BioRender.

Tissue-resident macrophages develop within the embryo and the adult mouse in a series of sequential developmental waves (**Figure 2**). At embryonic developmental day (E)8.5, erythro-myeloid progenitors (EMPs) in the yolk sac give rise to pre-macrophages (pMacs), which colonize the embryonic tissue (2, 3). These pMacs are present within the tissues during early development and differentiate into tissue-resident macrophages in parallel to organogenesis, thereby establishing cellular cross-talks with neighboring cells already early in life (4–6). In addition, at E10.5, hematopoietic stem cells (HSCs) emerge within the aorta-gonado-mesonephros (AGM) region from where they migrate to the fetal liver at E11.5 (7–10). There they expand and produce different lineages of leukocytes, and then finally migrate to the fetal bone marrow shortly before birth. At postnatal stages, bone marrow HSCs give rise to circulating monocytes, which can migrate into tissues and differentiate into monocyte-derived macrophages (MdMs) (4, 10). Albeit a fetal liver monocyte origin has been proposed for macrophages, there is to date no direct experimental evidence in mice showing that HSC-derived monocytes differentiate into macrophages during embryogenesis. Indeed, the fate-mapping mouse model *Ms4a3^Cre^; Rosa26^tdTomato^* specific to granulocyte-monocyte progenitors (GMP) has demonstrated that no tissue-resident macrophages are GMP-derived at birth (11).

A plethora of work has demonstrated that yolk sac-derived tissue-resident macrophages can proliferate and self-maintain within the tissue for months or even years (1). Using diverse tamoxifen-inducible fate-mapping models, it has been suggested that, with age, yolk sac-derived cells cease to proliferate and can be replaced by MdMs (12). The rate of replenishment by MdMs varies greatly between tissues; the tissue-resident macrophages of the brain parenchyma, microglia, self-maintain throughout life with little to no replenishment by MdMs; on the other end, macrophages in the gut lamina propria display a high turnover and are rapidly replaced by MdMs (reviewed excellently elsewhere) (1, 13). However, we would like to highlight that it has not yet been established whether MdMs that are starting to seed developing tissues after birth can become long-lived and proliferative as their yolk-sac derived counterparts (**Figure 2**). In any case, every tissue contains a mix of yolk sac-derived and monocyte-derived macrophages which dynamically changes over time, or as a consequence of inflammation or infection.

The distinct longevity of yolk sac-derived macrophages and MdMs highlights an intriguing aspect of developmental programming (**Figure 2**). Indeed, the first long-lived macrophages, such as microglia and Kupffer cells, are present as early as E9.5-E10.5 (3). Consequently, perturbations occurring during these early developmental stages can have long-lasting effects, as these cells proliferate *in situ* and give rise to adult macrophage clones that retain the information experienced by the progenitor macrophages. Conversely, developmental programming can also affect HSCs, which, in adulthood, give rise to MdMs. The impact of these perturbations depends on their duration and nature. Consequently, developmental disturbances can lead to varied outcomes based on the type of perturbation and the specific stage of development at which it occurs.

## 3 Niche programming vs. developmental programming

Once macrophages have colonized their tissue of residence, local cues determine macrophage longevity, identity and function. For instance, the expression of colony-stimulating factor 1 (CSF1) and IL-34 is essential for the self-maintenance of different macrophage populations (14). Further, niche programming constitutes factors such as IL-13 and colony-stimulating factor 2 (CSF2) for alveolar macrophages, transforming growth factor beta (TGFb), bone morphogenetic protein 9 (BMP9) and desmosterol for tissue-resident macrophages of the liver, Kupffer cells (15, 16). These niche programming events have been mostly studied during adulthood using macrophage depletion models. However, during development the intercellular interactions of a macrophage may be completely different than what we observe during steady-state adulthood. One such example is Kupffer cells, which act as primary macrophages for erythroblasts during embryogenesis, facilitating their maturation (17, 18). Additionally, these cells interact with fetal liver hematopoietic stem cells, thereby regulating their differentiation potential (19, 20). At this early time point, there are no hepatocytes present that could imprint Kupffer cell identity and drive gene expression of core Kupffer cell factors, such as Id3, which can already be detected as early as at E10.5 (21). Only from E14.5 onwards hepatocytes differentiate and directly interact with Kupffer cells (21). Finally, during the first weeks of life, Kupffer cells migrate to the lumen of sinusoids where they start to actively sample blood components and circulating cells (22). Another example is that of microglia, the tissue-resident macrophages of the brain. Indeed, it has been well-characterized that microglia participate in neuronal development early in life, and then switch to a neuro-supportive function in adulthood (23–25). It is probable that all long-lived macrophages undergo similar dynamic alterations in their cellular interaction partners during embryogenesis. We hypothesize that these interactions are essential for beneficial developmental programming of yolk sac-derived macrophages, enabling them to fulfill their tissue-specific functions necessary for maintaining tissue homeostasis later in life.

Beyond direct interactions, environmentally encoded developmental triggers, such as the maternal microbiota and food intake, may be essential for proper macrophage function. These factors, which may be interlinked, can lead to a state of chronic low-grade inflammation that can impact the developing fetus. However, also acute stressors during gestation may play a role, as has been demonstrated for stress and immune activation via, for example, bacterial or viral infections. The timing of these perturbations may also play a role in determining the outcome; indeed, while chronic stress, such as obesity, impacts all stages of development, acute stress may have a more restricted impact depending on when it occurs. Altogether, the core functions of adult macrophages are likely influenced by prenatal and early-life perturbations, which can have lasting impacts on macrophage precursors, including EMPs and pMacs, as well as on maturing macrophages, potentially through epigenetic mechanisms. However, developmental programming may have beneficial or detrimental outcomes, depending on the stimuli. From an evolutionary perspective, early-life or maternal priming of immune cells confer advantages for the growth and survival of offspring. For instance, measle-containing vaccines are associated with lower mortality in the first years after birth. Maternal priming has also demonstrated its importance in this context, as children with maternal antibodies from vaccination against measles have shown lower mortality rates (26). However, not all stimuli result beneficial outcomes (see below). Given the crucial role of macrophages in tissue maturation, their developmental programming can profoundly impact tissue function and homeostasis. This concept aligns with Barker’s theory of the “*Developmental Origin of Health and Disease*,” which suggests that environmental exposures during development can determine health and disease risk in adulthood. Of note, in addition to programming macrophage progenitors, developmental perturbations may also reprogram niche cells, which in turn, could impact macrophage function. Conversely, developmental reprogramming of macrophages may be masked by niche programming, which displays remarkable resilience and plasticity to external perturbations (1). The precise target of developmental programming may not always be clear throughout studies. However, as the topic of macrophage programming by the niche has been excellently covered elsewhere (27), and direct studies investigating the combined influence of developmental and niche programming are still lacking, we will here focus on how developmental perturbations may impact macrophages directly.

The nature of developmental perturbations affecting offspring immunity can be considered a regional issue. Indeed, in countries of the Global South, bacterial and viral infections, and accompanying antibiotic treatment, during gestation are far more common than those of the Global North (28–30). However, the exponential increase in obesity and metabolic disorders in all regions of the world poses a novel and unprecedented challenge, as these disorders have shown to have wide-ranging consequences on offspring immune function (31). Understanding the consequences of harmful developmental programming is of fundamental importance to consider its corresponding socioeconomic impact and to develop targeted interventions to safeguard the health of future generations.

Finally, while most studies focus on the maternal contribution to offspring immunity, increasing evidence suggests that developmental programming can also be transmitted from the paternal side via the sperm epigenome (32–34). However, in the following review we will focus on the maternal impact on offspring macrophage programming, highlighting current knowledge about maternal immune activation (MIA) associated with stress and infections, diet, as well as the role of maternal microbiome. An overview of models available to study the impact of developmental programming on the offspring is shown in **Table 1**.

**Table 1:** Observed macrophage phenotypes and functions after a trigger.

Observation	Animal Model	Prenatal Trigger	Macrophage population	Offspring Sex affected	Comment	Reference
Diet						
Increased TNFa in response to LPS	Mouse	HFD	Microglia	Male		(35)
Increased phagocytosis of serotonin	Mouse	HFD	Microglia	Male	In the dorsal raphe nucleus	(35)
Altered cell morphology	Mouse	HFD	Microglia	Male	In the hippocampus	(36)
Increased cell density	Non-human primate	HFD	Microglia	Not specified	In the basolateral amygdala	(37)
Decreased cell density	Mouse	High fructose diet	Microglia	Not specified	In the prefrontal cortex	(38)
Decreased phagocytosis and efferocytosis	Mouse	High fructose diet	Microglia	Not specified		(38)
Pro-inflammatory cytokine expression	Non-human primate	WD	Kupffer cells	Not specified		(39)
Increased pro-inflammatory gene expression in response to LPS	Non-human primate	WD	BMDMs	Not specified		(40)
Increased glycolysis and reduced oxidative phosphorylation	Non-human primate	WD	BMDMs	Not specified		(40)
Increased phagocytosis	Non-human primate	WD	BMDMs	Not specified		(40)
Reduced cytokine production	Non-human primate	WD	Intestinal macrophages, Splenic macrophages	Not specified		(41)
Microbiota						
Higher microglia density (Embryo)	Mouse	Disbyosis, GF mice*	Microglia	Male	At 14.5 and 16.5. In somatosensory cortex, striatum and preoptic area	(42)
Higher microglia density (Postnatal)	Mouse	Disbyosis, GF mice*	Microglia	Female	At P20. In somatosensory cortex, striatum and preoptic area	(42)
Transcriptomic alterations	Mouse	Disbyosis, GF mice*	Microglia	Male and Female	In embryonic stages more pronounced in males, but postnatally in females	(42)
Changes in chromatin accessibility	Mouse	Disbyosis, GF mice*	Microglia	Male and Female	Females display more differences.	(42)
Impaired synaptic engulfment	Mouse	Dysbiosis, GF mice**	Microglia	Male and Female	In the cerebellum	(43)
Maternal Immune Activation						
Increeased microglial process tip speed	Mouse	PolyI:C	Microglia	Not specified	At E18 and adulthood if induced at E12, resolved at P10 if induced at E15.	(44)
Upregulation of pro-inflammatory cytokines	Mouse	PolyI:C	Microglia	Not specified	At E18 and adulthood if induced at E12, resolved at P10 if induced at E15.	(44)
Transcriptional changes	Mouse	PolyI:C	Microglia	Not specified	At E18 and adulthood if induced at E12, resolved at P10 if induced at E15.	(44)
Reduced phagocytic capacity	Mouse	PolyI:C	Microglia	Male	In the hippocampus	(45)
Altered cytokine secretion at baseline and following LPS	Mouse	PolyI:C	BMDMs	Male and Female		(46)
Increased cell density	Mouse	Influenza A	Cecal myeloid cells	Not specified		(47)
Increased cell density	Mouse	Influenza A	Border-associated macrophages	Not specified	In the Pia Mater	(48)
Maternal Stress						
Increased expression of CCL3 and CCL4	Mouse	Cold stress	Microglia	Male	In the hypothalamus	(49)
Accelerated differentiation in early postnatal stages	Rat	Forced swim test	Microglia	Not specified	In the corpus callosum, internal capsule, septum, amygdala, thalamus, entorhinal and perietal cortices. It is normalized at P10.	(50)
Reduced number of immature microglia in newborns	Rat	Forced swim test	Microglia	Not specified	In the corpus callosum, internal capsule, septum, amygdala, thalamus, entorhinal and perietal cortices. It is normalized at P10.	(50)
Higher microglia density	Rat	Sleep deprivation	Microglia	Male	In the hippocampus	(51)
Altered morphology, shorter and fewer microglia processes	Rat	Sleep deprivation	Microglia	Male	In the hippocampus	(51)
Impaired resorptive capacity of osteoclast	Rat	Constant light	Osteoclast	Male	In the dental area	(52)
Pollutants						
Increased microglial density	Rat	Nanoplastic (50nm, anionic)	Microglia	Not specified	In the cortex	(53)
Impaired synaptic pruning	Mouse	Diesel exhaust + maternal stress	Microglia	Male	In the anterior cingulate cortex	(54)

Summary of the observations following prenatal exposure to different triggers, and their corresponding models. HFD: High-Fat Diet. WD: Western Diet. GF: Germ-Free. * In these settings, the microbiota of GF mice is compared to SPF conditions. **In this study, GF mice were compared to mice colonized with a consortium of Bifidobacteria or a simplified consortium.

## 4 Potential triggers of developmental programming

### 4.1 Diet

Research on maternal diet has focused mainly on maternal high-fat diet (HFD) or maternal ‘Western-style’ diet (WD). While both diets are rich in fat, WD is also high in sucrose, whereas HFD has a sucrose content comparable to a regular chow diet. However, drawing parallels across different studies is hampered by the specific composition of the maternal diet, e.g., the level of fat content and the source of dietary fat (corn oil, peanut oil, soybean oil, sunflower oil, or lard), which can lead to microbiota perturbations and subsequent downstream effects (55). Despite these challenges, it is widely accepted that maternal obesity, regardless of the specific diet, leads to alterations in the offspring’s immune system, particularly affecting macrophage responses to inflammatory stimuli. Microglia and Kupffer cells may represent key targets of developmental programming through the maternal diet. Indeed, both microglia and Kupffer cells are seeded early during embryonic development, and receive very little input from circulating cells throughout life. As such, information programmed in these cells during early development can have long-lasting effects on tissue function. Nevertheless, while a large volume of studies has focused on the impact of maternal diet on offspring microglia, much less is known about resident macrophages in other tissues.

There is compelling evidence suggesting that maternal obesity has a profound impact on neurodevelopment through alterations in microglia. These effects are notably sex and region-specific. For instance, microglia from male embryos of mothers fed a HFD showed increased TNFa production in response to LPS (35). Moreover, maternal HFD leads to increased CD68 immunoreactivity and increased phagocytosis of serotonin by microglia in the dorsal raphe nucleus of male offspring. This results in decreased serotonin availability in both fetal and adult brains, contributing to sex-specific behavioral outcomes. These changes were absent in a *Cx3cr1^CreERT2^; Tlr4^fl/fl^* conditional knockout model, implicating TLR4 signaling in these processes. This study further found increased endotoxin levels in placenta and fetal tissue of HFD-exposed offspring compared to offspring born to control diet (CD)-fed mice, suggesting that the adverse effects of maternal HFD on neuronal function are partly mediated by increased endotoxin and TLR4 activation (56). This evidence supports the hypothesis that the inflammatory environment induced by maternal obesity may contribute to the higher incidence of neuropsychiatric disorders, such as ADHD, in male offspring (57).

In mice, the dorsal hippocampus of juvenile offspring from HFD-fed mothers displayed altered microglial morphology, while overall microglial count remained unchanged (36, 58). In non-human primates, juvenile offspring of HFD-fed mothers displayed increased microglial numbers in the basolateral amygdala (37). While the functional consequences of increased microglial density remain to be elucidated, these findings suggest that maternal obesity can significantly disrupt microglia homeostasis in offspring, potentially affecting neurodevelopment.

While the consequences of maternal obesity on neurodevelopment have been well-characterized, emerging evidence suggests that other maternal dietary preferences may also have a profound effect on offspring brain development. A recent preprint highlights how maternal high-fructose diet led to decreased microglial density, decreased synaptic pruning, and deficient efferocytosis, which was reflected by an increase in uncleared apoptotic cells. Consequently, changes in microglia core functions lead to cognitive defects and anxiety-like behavior in the offspring. This was mediated via the high affinity fructose transporter SLC2A5 (GLUT5), as its deletion in neonates completely reversed microglia dysfunction and cognitive defects (38). Similarly, a recent study evidenced that maternal high-salt diet could affect synaptic plasticity and memory in the offspring, however it is unclear if these effects were mediated by microglia (59). Taken together, microglia clearly harbor the capacity to memorize cues from the maternal diet during gestation, which can then have profound consequences on the offspring neurodevelopment.

Kupffer cells also represent an interesting candidate for developmental programming by maternal diet. Indeed, Kupffer cells are predominantly fetal-derived and are only replaced very slowly by monocyte-derived macrophages during steady-state conditions (6). In line, in a non-human primate model it has been shown that a maternal WD leads to increased liver macrophage pro-inflammatory cytokine expression (Il-1b, TNFa, Il-6) in the offspring (39). It is intriguing to speculate that this developmental programming may contribute to the increased prevalence of fatty liver disease observed in the offspring of overweight mice, and potentially, humans (60–62).

Finally, while alterations in the long-lived resident macrophage populations can lead to developmental changes during tissue development, maternal obesity also impacts the progenitors of circulating monocytes in the bone marrow, which increasingly colonize tissue-resident macrophage niches as the organism ages. These changes have been observed to occur already in progenitors present in the fetal liver, which can lead to permanent changes in the bone marrow that persist throughout life. In mice, maternal WD leads to remodeling of fetal HSCs, skewing toward myeloid and B cell differentiation over HSC self-renewal (63). Similarly, in non-human primates, maternal WD induces transcriptional reprogramming of HSCs in the fetal liver, with upregulation of pathways involved in TNF signaling, oxidative phosphorylation and antigen processing and presenting (64). Of note, these transcriptional changes in HSCs from maternal obese offspring were epigenetically imprinted (40). In line with these findings, bone marrow-derived macrophages (BMDMs) differentiated from adult offspring of WD-fed non-human primates presented heightened pro-inflammatory gene expression (Il-1b, TNF) following LPS stimulation. This was accompanied by increased glycolysis, reduced oxidative phosphorylation, and increased phagocytosis of fluorescent bioparticles (40). These changes can impact tissue homeostasis as this cellular reprogramming may also occur *in vivo* in MdMs that increasingly colonize tissues. However, the biological consequences of such reprogramming remain unclear and may affect specific organs differently. An organ of particular interest regarding the functional consequences of developmental HSC programming is the intestine, where macrophages undergo high turnover by MdMs. Intriguingly, in a non-human primate model of maternal WD, offspring macrophages displayed a reduced production of TNFa and CCL20 and decreased capacity to respond to bacterial stimulation (41). Similarly, splenic macrophages, which also display turnover by MdM, also displayed reduced secretion of TNFa and IFNa in offspring from maternal obese mothers (41).

Taken together, maternal diet can have a significant impact on offspring immunity. The functional consequences of such developmental programming differentially impact fetal-derived macrophages and MdM, which can thus impact tissue homeostasis on different timescales and contexts. Further studies are warranted to fully elucidate the mechanisms involved.

### 4.2 Microbiota

Microbiota is a collective term to refer to the consortia of microorganisms that reside within an individual. The microbiota profits from the host’s environment, and in return, provides the host with advantages, such as protection against pathogens, digestion of otherwise undigestible dietary components and the production of dietary metabolites (65). Additionally, the microbiota is known to modulate the host’s immune system. Remarkably, commensal-derived metabolites contribute to fetal development and can directly shape the immune system of both mother and offspring. It is well-known that, beyond the maternal gut microbiota in prenatal stages, exposure to the vaginal and skin microbiota during birth or lactation is crucial for the correct priming of the immune system. In human babies born from vaginal delivery (VD), the infant’s microbiome is rich in Bifidobacteria, Lactobacillus, and Bacteriodotes and reduced in Enterococci and Klebsiella, in comparison to caesarean section-born infants. Microbiome in mouth, meconium and nose of C-Section (CS) babies resembled the mother’s skin, while VD babies harbored commensals that resembled more the mother’s vaginal microbiota (66). Interestingly, CS infants need about one year to achieve the microbiota baseline of their VD counterparts (67, 68). CS has long-term impacts on health since it has been linked to immune-system dysregulation and immaturity and higher risk of non-communicative diseases like type-1-diabetes, allergies, and obesity (69–72). Despite extensive descriptions of the impact of vaginal microbiota exposure and long-term health of offspring, the concrete influence on macrophages remains unknown.

Typical environments for studying the influence of microbiota in animal models include the use of germ-free (GF) mice, which are devoid of microorganisms. When mice are inoculated with specific, known bacterial strains they are termed gnotobiotic. The gnotobiotic setup allows researchers to study the roles of individual bacterial strains (monocolonization) or defined consortia of microorganisms (73). Specific-pathogen-free (SPF) mice, which have more heterogeneous microbiota, can vary between facilities and among individuals or communities within them (e.g. cages). Microbiota manipulation can also involve the usage of antibiotics or cohousing with animals harboring different commensals, such as pet shop mice, wild mice or wildlings (74). Taken together, these tools can be leveraged to assess the impact of the microbiota on individual tissues or specific cell types. One limitation to be noted in the context of early-life exposure is that GF mice develop and are born under GF condition, which makes it difficult to elucidate if observations result from prenatal or postnatal exposures, or from both. It should also be taken into consideration that these mice are often maintained throughout generations in GF facilities, so it is unclear whether observed phenotypes relate to gestation, or to the cumulative effect of several generations in the absence of microbiota.

In the brain, studies comparing GF and SPF embryos revealed an impaired microglial density at E14.5, E16.5 and postnatally in both the somatosensory cortex and the striatum. These observations were sex- and age-specific; males exhibited higher microglial density during embryonic stages while females showed higher density postnatally (42). Further analysis using ATAC-seq on GF and SPF embryos and adults revealed a different chromatin accessibility. Overall, SPF embryos displayed increased accessibility in regions related to core microglial functions and LPS responses (42, 75). The differentially accessible regions were mainly related to microglial functions and LPS responses.

A study published by Luck et al. focusing on post-natal mice during the first to third week of life identified a crucial link between early-life commensals and microglia in neurodevelopmental processes (43). The study involved mice born under GF conditions that were subsequently either maintained as GF or exposed to colonization with either a Bifidobacterium species or a conventional microbiota. The absence of a microbiome resulted in impaired synaptic engulfment of cerebellar Purkinje cells by microglia, a process crucial for proper neuronal maturation and function. This caused an impaired firing rate of the Purkinje cells, as determined by electrophysiology. Additionally, microglia from GF conditions were fewer in number and displayed lower expression levels of CD68 and CD36 in the postnatal stages. CD68 is a glycoprotein associated with phagocytosis, and CD36, although primarily known as a fatty acid transporter, plays a role in the phagocytosis of apoptotic cells and modulation of inflammatory responses (76). Altogether, these observations indicate that exposure to microbiota in this early period of development significantly influences microglial function.

The microbiome can synthesize essential vitamins *de novo* and produce short-chain fatty acids (SCFA) through fermentation of dietary fibers (77, 78). Among SCFA, acetate is defined as the most important in modulating the maturation, morphology, and function of microglia (79, 80). Mice deficient for SCFA receptors displayed microglia phenotypes of GF mice (80). Furthermore, administration of these metabolites in GF mice restored microglia density, maturity, and morphology (80). Studies show that maternal acetate and other SCFA can cross the placenta and influence the acetate levels of the offspring but it is not clear through which mechanisms they can influence developing microglia or other tissue-resident macrophages (81, 82). A leading hypothesis suggests that, as microglia fail to express Gpr43 and Gpr410 (G protein-coupled receptors sensitive to SCFAs), cytokine signals from peripheral cells harboring those GPCRs influence the epigenetic regulation of microglia (83). Interestingly, a recently published article showed the influence of the SCFA butyrate in the process of efferocytosis (84). In the study, mice raised in GF conditions or treated with antibiotics exhibit a decreased clearance of apoptotic cells compared to SPF mice. Although this study was performed in adult peritoneal macrophages, and the phenotype of antibiotic-treated animals was rescued by fecal transplants, it remains crucial to elucidate the consequences of impaired efferocytosis or other core macrophage functions specifically during development.

Taken together, it is clear that the microbiota can influence immunity in the offspring. However, it should be noted that diet can profoundly influence the microbiota, suggesting that these two developmental triggers are interlinked. Indeed, WD has been shown to reduce microbial complexity, thereby reducing production of SCFAs including acetate (85). Furthermore, while microglia serve as perfect candidates to study the microbiota’s effects on long-term developmental programming, studies have also highlighted changes occurring in other tissue-resident macrophage subsets, including the gut, the spleen and the liver (86–88). However, most of these changes are normalized following commensal recolonization, suggesting they reflect a direct response to the presence or absence of commensals, rather than developmental programming of macrophages themselves. These findings further highlight the resilience and plasticity of the niche, which is able to restore macrophage function upon cessation of the perturbation. Further studies should focus on teasing apart these mechanisms.

### 4.3 Maternal immune activation

Maternal immune activation (MIA), an inflammatory event occurring during gestation, has been linked to neurodevelopmental disorders and increased prevalence of psychiatric disorders later in life (reviewed in (89)). As microglia develop exclusively from YS progenitors early in development, and persist throughout life via self-maintenance, these cells are of particular interest in the context of developmental programming, in particular due to their role in supporting neuronal development and connectivity early in life (reviewed elsewhere recently (90)). It is now widely accepted that MIA leads to microglial activation, transcriptional changes, and functional consequences in the offspring, however, the lack of standardization across studies hampers study interpretation. In particular, gestational timing of MIA induction, the gender of the offspring and the severity of the inflammation have been shown to determine study outcomes (reviewed in (91)). In addition, the method of MIA induction also varies across studies; most commonly used are LPS, to mimic a bacterial infection, and PolyI:C a synthetic analog of double-stranded RNA (dsRNA), which mimics a viral infection. However, several authors have directly employed administration of pro-inflammatory cytokines during gestation to mimic the consequences of MIA. Finally, bacterial or viral infections can also be employed to induce MIA. With regards to the latter, evidence is accumulating that MIA may also impact other tissue-resident macrophage populations in the body, as detailed below.

#### 4.3.1 PolyI:C

MIA has been shown to induce changes in microglial gene expression, function and motility in the offspring. Indeed, MIA induced at E12 or E15 results in increased microglial process tip speed in the offspring at E18, as well as in response to LPS challenge in adulthood (44). Process motility is a physiological function that allows microglia to constantly sample the environment and respond to potential stimuli, therefore these changes may reflect a reduced capacity of microglia to respond to neuronal needs and potential damage. These changes were accompanied by upregulation of pro-inflammatory cytokines, in particular IL-6, in fetal microglia following MIA. With regards to microglial motility and behavioral outcomes, MIA at E12 induces deficits that persist into adulthood, while MIA at later gestational stages results in transient alterations, most of which resolve by postnatal day 10 (44). However, MIA at E15 can still induce transcriptional changes in hippocampal microglia, which persist into adulthood, in particular upregulation of genes involved in inflammatory response, cell migration, and phagocytosis. In line, adult microglia from E15 MIA offspring display reduced phagocytic capacity (45). Of interest, the authors demonstrated that the transcriptional changes, which were accompanied by behavioral deficits, could be partially restored by postnatal treatment with minocycline, suggesting that developmental programming of microglia may be reversible and could be an interesting therapeutic target in psychiatric disorders (45).

Of particular importance in the effects of MIA on microglia is IL-6, as IL-6 knockout mice or the administration of an IL-6 neutralizing antibody show ameliorated microglial outcome following MIA (92). However, IL-6 blockade does not rescue behavioral defects in adult MIA offspring, suggesting that other mechanisms may also be involved (93). In line, Yu et al. recently demonstrated that MIA induces downregulation of Gpr56 in microglia, and that microglia-specific Gpr56 abrogation phenocopied the neurodevelopmental defects and autism-like behaviors observed in MIA offspring. Conversely, microglia-specific upregulation of Gpr56 was able to ameliorate behavioral deficits in MIA offspring (94). The critical role of microglia in mediating the neurodevelopmental effects of MIA in the offspring is further highlighted by a study by Ikezu et al., that demonstrated that behavioral deficits and synaptic abnormalities were abrogated by depleting microglia postnatally in the offspring (95).

The consequences of MIA on microglia have been well-documented, however whether other tissue-resident macrophage populations are affected is hitherto unknown. Of interest, MIA can also alter the immune response of bone-marrow derived macrophages in the offspring. In a murine model of maternal polyI:C injection, BMDMs obtained from PolyI:C exposed offspring had altered cytokine secretion at baseline and following LPS stimulation (46). Further studies are warranted to fully elucidate the consequences of MIA on peripheral tissue-resident macrophages and HSCs of the offspring.

#### 4.3.2 Influenza A

Influenza A virus (IAV) infection during pregnancy has been shown to increase the risks of preterm birth, low birth weight, and even infant mortality, suggesting that gestational viral infections can have profound consequences on the offspring (96). However, in humans, it is not clear if these adverse outcomes are a direct result of altered offspring immunity. In mice, offspring born to mothers infected with IAV during gestation were more susceptible to heterologous infections compared to offspring from control or polyI:C-treated mothers. These effects were partly mediated by the imprinting of alveolar macrophages; transfer of alveolar macrophages from offspring of control mothers improved disease recovery and clearance of viral titers, despite an increased frequency of alveolar macrophage in maternal IAV offspring. Mechanistically, IAV infection during gestation altered hematopoiesis in the bone marrow of the offspring, leading to increased numbers of HSCs, c-Kit+ progenitors, and common myeloid progenitors (CMPs) in maternal IAV offspring compared to control or polyI:C-treated offspring (97). These alterations in hematopoiesis might also explain the increase in myeloid cells in the caecal patch, a large lymphoid aggregate in the intestine, in offspring from IAV-infected mothers compared to controls (47). However, the biological relevance of the increased myeloid cells in the cecal patch remains unknown. Finally, it has been shown that maternal IAV infection can lead to increased numbers of border-associated macrophages (BAMs) in the pia mater of the offspring, while microglial numbers remained unchanged (48). While the functional consequences of this expansion are unknown, it is intriguing to speculate that these cells, which lie at the interface between brain and periphery, may expand to protect the developing brain from peripheral danger (98).

### 4.4 Prenatal stress

The term ‘prenatal stress’ refers to psychological or physiological stressors experienced during pregnancy that can affect the developing fetus. In response to such challenges, the body activates its stress response systems, which help adjust to demands by mobilizing energy, modulating the immune system, and promoting behavior and cognition to improve survival (99–101). The stress response systems include the sympatho-adrenomedullary and the hypothalamic pituitary adrenal gland (HPA) axis (102, 103). While the sympatho-adrenomedullary system is responsible for rapid, acute responses, we will focus on the HPA axis, which is decisive for understanding the effects of chronic stress.

One of the most common prenatal stressors is related to disordered maternal nutrition, either through food restriction or excess, which was described in more detail in the diet chapter (102, 104–106). Other known stressors include the disruption of the maternal circadian rhythm and the usage of artificial glucocorticoids (107–109). Additionally, traumatic life experiences that are related to depression and anxiety can also impact the offspring (110). Population studies have reported that infants exposed to maternal psychological stress during pregnancy are at higher risk for infection-related hospitalizations, with this risk being higher in boys (110, 111).

Stress arises from the inability to adapt to stressors, leading to chronic activation of the HPA axis and prolonged exposure to glucocorticoids, which can be detrimental to offspring (101, 112). Glucocorticoids (corticosterone in rodents, fish, and amphibians, and cortisol in humans and guinea pigs) are essential hormones for the stress response and various physiological processes (113). They play a crucial role in the development of prenatal and postnatal organ maturity (114) and are instrumental in the maturation of the cardiovascular and immune systems (103, 115, 116). As birth approaches, glucocorticoid levels gradually increase to support the metabolic demands of the mother (117). However, imbalanced and prolonged glucocorticoid exposure can adversely affect offspring prenatally by altering cardiovascular functions and the immune system, thereby increasing the risk of metabolic and psychiatric diseases (102, 112, 118, 119).

The placenta and the fetus are equipped with mechanisms that protect from maternal glucocorticoids, primarily through the expression of the enzyme 11ß-hydroxysteroid dehydrogenase type 2 (11ß-HSD2), which breaks down active glucocorticoids (120). In humans, this enzyme is expressed from the 5^th^ week of pregnancy in the placenta and it is effective in neutralizing most maternal glucocorticoids (121). Nevertheless, this mechanism cannot cope with prolonged, high levels of the hormone, or with synthetic glucocorticoids, which could be metabolized less efficiently than endogenous glucocorticoids.

Prenatal stress in mice is modeled using various methods. Different stressors applied to pregnant dams include light stress, sleep deprivation, restraint, exposure to foot shock, cold stress, and the forced swimming test (49–51, 122). These different stress paradigms share the common feature of inducing measurable maternal hormonal changes that can be transferred to the fetus. As discussed in previous sections, microglia are ideal candidates for developmental programming due to their long lifespan. Consequently, most studies have focused on these cells and the impact of their reprogramming on neurodevelopment. Microglia can sense stress through receptors sensitive to glucocorticoids, such as NR3C1 and NR2C2, whose transcripts have been detected in embryonal stages (123, 124). One proposed mechanism, described in postnatal microglia, indicates that these cells exhibit pro-inflammatory responses upon exposure to glucocorticoids (124, 125). Although glucocorticoids are typically described as immunosuppressive, they can also exert pro-inflammatory effects through the so-called “permissive effect” when administered in basal doses (125, 126).

In a model of prenatal stress using a forced swim test, pregnant rats subjected to forced swimming exhibited a reduction of immature microglia and accelerated differentiation in the newborn brains of their offspring, as indicated by an increased number of ramified microglia. This effect normalized after 10 days of life (50). Similarly, prenatal stress induced by sleep deprivation and other stressors has been shown to alter microglial density and induce region-specific morphological changes in microglia (51, 127–129). In the hippocampus, maternal sleep deprivation resulted in decreased and shorter microglial processes, along with reduced neurogenesis. Interestingly, the administration of pioglitazone in adolescent mice mitigated cognitive deficits by enhancing PPARγ-dependent microglia-mediated neurogenesis, suggesting that the developmental reprogramming of microglia by prenatal stress may be partially reversible (51). Furthermore, in the hypothalamus, Rosin et al. demonstrated that ventricular-residing microglia respond prenatally to stress in a model of maternal cold stress (49). Maternal cold stress affected neuronal specification and glial fate in a sex-dependent manner, impacting male but not female brains. This effect was reversed by the removal of microglia with the PLX5622 diet, highlighting the critical role of microglia as intergenerational messengers. Interestingly, a recent study in adult mice showed that a foamy-like macrophage within the adrenal gland arises from chronic and acute stress (130). This macrophage subtype can modulate glucocorticoid production by restricting steroidogenesis via Trem2 and TGF-β. Giving the long-lived nature of macrophages and their capacity to react timely to exposures, it would be intriguing to investigate whether developmental programming may impact adrenal macrophages, which thereby can directly modulate systemic stress responses.

Epigenetic changes represent another possible mechanism of action in response to prenatal stress. A meta-analysis of independent human longitudinal studies associating prenatal stress with newborn DNA methylation revealed that abuse during pregnancy was linked to methylation of CpG regions, such as those on chromosome 7 annotating the Myeloid Differentiation Primary Response 88 (*MYD88*) gene (51). This gene, related to immune system function, is also crucial for the development of the central nervous system. Nevertheless, further studies are required to elucidate these effects and determine whether they have a direct impact on macrophage development and function.

Finally, one study focused on osteoclasts, which are highly specialized bone-resident macrophages (52). Osteoclasts, derived from EMPs at birth, are responsible for bone resorption by dissolving bone minerals (131). This process generates the bone marrow cavity and facilitates tooth eruption (131). Fontanetti et al. induced stress in pregnant rats by exposing them to constant light during the last 10 days of gestation. This exposure resulted in impaired bone remodeling and reduced osteoclast activity in the dental area and impaired tooth eruption in the offspring born to stressed mothers (52). In conclusion, while prenatal stress significantly impacts fetal development by altering immune responses, microglial function, and bone remodeling, future studies are needed to elucidate the precise mechanisms and long-term effects of these changes, particularly regarding the role of epigenetic modifications.

### 4.5 Pollutants

Environmental pollutants also play a significant role in shaping the developing immune system. Multiple studies have demonstrated that a large number of foodstuffs destined for human consumption are contaminated with plastic particles smaller than 5mm, known as micro- and nanoplastics (132–136). While the impact of micro- and nanoplastic exposure on human health is just beginning to be elucidated, the effects of these contaminants on macrophage programming during development remain unclear. Of note, recent studies found microplastic contamination in all human placental samples analyzed, and in 75% of all breast milk samples, suggesting that these contaminants may pose a hitherto unknown risk to offspring health and immunity (53, 137). Although rodent studies of gestational microplastic exposure are limited due to significant differences in placental structure between humans and rodents, they may offer insights into how microplastics affect offspring immunity and development indirectly by causing immune activation in the placenta. For instance, a recent study in rats showed increased microglial density in the offspring of microplastic-exposed mothers compared to controls, accompanied by behavioral changes (138). Given the size-dependent capacity of plastic particles to pass the human placental barrier, it is imperative that future studies clarify the impact of micro- and nanoplastics on offspring immunity, especially in models that more accurately mimic the human fetal-placental interface (139).

Similarly, murine models of gestational air pollution exposure have shown region- and sex-specific alterations in microglial density (54, 140). A recent study combining diesel exhaust particle exposure and maternal stress demonstrated profound behavioral changes in male offspring only, accompanied by impaired microglial pruning during postnatal development in the anterior cingulate cortex (141). These findings suggest that individual environmental triggers can have a cumulative effect on offspring macrophage function and overall immune development.

## 5 Concluding remarks

Macrophages originating from progenitor cells that arise from the yolk sac, colonize the tissues during development and persist through self-maintenance. Both local and developmental cues shape their identity, longevity, and function. Macrophages play an important role in homeostasis, contributing to the active regulation of their environment, but also in allostasis, by preparing the individual for forthcoming challenges later in life. For this reason, developmental programming could be understood as either beneficial or detrimental depending on the stimuli, their intensity, and the time window in which they appear. Additionally, individual factors including offspring gender, duration of the stimuli, and nature of the stimuli significantly impact outcomes.

While substantial evidence indicates that early development is a critical moment for programming of cellular responses, more research is needed to identify developmentally sensitive periods. Furthermore, more sex-inclusive studies are essential to uncover underlying mechanisms, as sex plays a significant role in determining immunological responses, macrophage function, and macrophage replenishment by recruited monocytes (142, 143). The microbiota, which can vary meaningly across mouse facilities, and certainly among human individuals, also plays a crucial role in shaping the immune system. It is important to recognize that the microbiota of laboratory mice does not reflect the natural microbiota of wild mice. Using lab-strain embryos transferred into wild mice or co-housing laboratory mice with wild mice (wildlings) will provide more accurate models for natural immune phenotypes (74). Upcoming studies on wildlings will enhance our understanding of macrophage development in natural environments.

Finally, recent evidence indicates that also environmental pollutants, such as micro- and nano-plastics, or air pollutants, during gestation may also impact offspring macrophage function. The impact of such exposures may be massive, and may disproportionately affect specific global regions more than others, highlighting the need to rapidly increase our understanding of the impact of these pollutants on the future generations, in order to accurately inform policies and global guidelines.

Understanding the origin of macrophages and the impact of maternal-derived developmental triggers is crucial for assessing the consequences and potential interventions to mitigate adverse effects. Future research using fate-mapper models will clarify macrophage origin and determine further whether certain stressors or their timing influence macrophage longevity and monocyte replacement in certain tissues. Consequently, macrophages can be considered intergenerational messengers, storing information received early in life, including during gestation, and thereby influencing health in adulthood and beyond. This perspective underscores the importance of safeguarding the period before and during pregnancy to create a favorable environment for offspring development.

In conclusion, while the evidence strongly supports the role of developmental programming in influencing macrophage function, we recognize that the local tissue environment and niche programming may also play a part. Although direct studies investigating the combined influence of developmental and niche programming are still lacking, we are confident that further research in this area will provide greater clarity. This review underscores the need to explore how both developmental cues and tissue-specific factors shape macrophage identity over time.
